# The Medical Work of the Local Government Board

**Published:** 1893-10-28

**Authors:** 


### The Medical Work of the Local Government Board.

### THE MANURE CONUNDRUM.

Manure is a nuisance; manure is a necessity. The town wants to get rid of it, the country wants to obtain it; apparently nothing could be more satisfactory, and in reality nothing is more conspicuously the reverse. For manure is offensive to human nostrils, if not dangerous to human health, and the question of how to convey it from city to soil is one of the conundrums that puzzle railway companies, sanitary officers, and authorities in general.

There can be no doubt that the carriage of manure is often most carelessly conducted. Town manure is most in demand in those localities where farming has given place to market gardening on a large scale, such as the Thames Valley and some parts of Bedfordshire. These largely supply the London market, and what are we to say when we learn that the same trucks take vegetables to town and come back to the country laden with manure! However much our cauliflower and asparagus may owe to the refuse of the city, it is not pleasant to think of their coming into contact with it in that fashion.

All manure is not, indeed, equally offensive, nor is it equally disagreeable at all stages of its decomposition. Fresh manure is not unendurable, thoroughly rotten manure is tolerable, but between these two extremes, when it is heating, the odour from it is loathsome, and there seems little doubt that its presence is mischievous. We say little doubt rather than none, because Dr. Parsons, who contributes a minute and voluminous report on the subject, though he found everywhere complaints of headache, sore throat, diarrhœa, and general malaise, among those who lived near sidings and other places where manure was stored, he found it impossible to discover any case of distinct disease where the general sanitary conditions of dwelling or district would not account for it, at least in part. And, indeed, the most general complaint is of nausea arising from the smell of manure, and it is not that which is most malodorous which is most harmful. For example, the most offensive effluvium arises from offal—the off-scourings of slaughter-houses, with condemned meat, unsaleable poultry, and refuse fish. But this is not by any means so dangerous to health as night-soil, that is, the refuse of food that has passed through the animal body. Yet so little is this understood that people will complain loudly of injury to their health from London manure, from which night-soil is excluded, being laid down in proximity to their houses, or even passing through their streets, while they tolerate at the same time the long presence of human excreta in a midden in their own back garden.

Yet, as we have said, there seems little doubt that the presence of manure is dangerous to health. When, as in one instance noted by Dr. Parsons at Dartford, among the refuse were seen soiled bandages, seemingly from a hospital, it requires no great imaginative power to think that disease may arise from its near neighbourhood. Moreover, while it is difficult to trace illness to the presence of manure in many instances, it has been reported by skilled observers, especially in the Eastern Counties, that outbreaks of diphtheria have been immediately preceded by the arrival of peculiarly offensive cargoes of London manure. Dr. Parsons noted also that men who were employed in loading manure from ships on to lighters and barges often seemed to suffer, while their families, living away from the scene of the work, were well. On the other hand, illness seemed to be the exception among the women dust sifters at Letts' Wharf. Of 499 who were examined, and who had been engaged in the work for periods varying from a month to twenty-five years, forty-one had never been laid aside except during their confinements, four had suffered from accidents, one from brain fever, one from pleurisy, two from "debility," and one from "pains across the stomach for six weeks." This exemption may be due in part to the greater toughness of the feminine constitution, but in part it may be credited to the fact that a great deal of offal is collected privately for various purposes, and does not come to the sorters of Letts' Wharf. Still, from the report regarding the manure depôt of the Newington Vestry at Manor Place, Walworth, it does not seem that the neighbourhood of large quantities of manure is invariably hurtful. The manure consists of road sweepings mixed with stable dung, and when Dr. Parsons visited the depôt on January 6th, 1892, there were between 4,000 and 5,000 tons lying there. Yet from the neighbourhood, which is a populous one, no complaint of nuisance was received, nor was there more fever or other sickness there than in other parts of the parish. It is considered that both danger and disagreeableness are diminished by leaving the manure to stand until it is thoroughly decomposed, instead of moving it while "heating" is going on.

But even if all danger were disproved the presence of large heaps of manure in populous places is a nuisance of the most objectionable nature. Yet in some places a disgusting indifference seems to prevail as to when and how it is removed to the ground it is to fertilise. The manure from foreign cattle ships, which is of a peculiarly offensive character, owing to the fermentation of the waste maize in it, was formerly obliged to be kept in the dock until the animals imported in the ships were slain, which did not necessarily occur before the end of ten days. An absolutely useless nuisance was thus made compulsory; but this error is now partly corrected, and the manure may be got rid of as soon as the Customs officer, with whom lies the power of keeping it in dock, is assured by the inspector that the animals who were on board the ship were not suffering from cattle plague, foot-and-mouth disease, swine fever, or sheep-pox, these being the only diseases conveyed by manure. When any of these diseases has been present the germs are destroyed by mixing the manure with its own weight of quick-lime. It is not then, however, used in agriculture; but is taken out to sea, and there deposited.

Complaints have often been made to the railway companies with regard to manure, and they have been asked to erect special sidings where it can be unloaded and stored; but naturally the directors object to such an unremunerative employment of capital, and the officials of the South-Eastern Railway protested that they would rather give up carrying manure than go to any expense. In view of the needs of the farmers, this alternative could not be entertained. The best that can be done is to insist that the farmers to whom manure is consigned should remove it at once from the station where it is unloaded, employing for the purpose good carts, covered with tarpaulin, so that offensive matter may not be dropped on the roads, and the effluvia may be prevented, as much as possible, from escaping. In a farmyard or a corner of a field it might lie decomposing till it was required without injuring comfort or endangering lives as it now does when stored for an indefinite time at railway sidings or on public wharves.

